# Engaging a Rising China through Neglected Tropical Diseases

**DOI:** 10.1371/journal.pntd.0001599

**Published:** 2012-11-29

**Authors:** Peter J. Hotez

**Affiliations:** 1 Sabin Vaccine Institute and Texas Children's Hospital Center for Vaccine Development, Houston, Texas, United States of America; 2 National School of Tropical Medicine, Department of Pediatrics and Molecular Virology & Microbiology, Baylor College of Medicine, Houston, Texas, United States of America; 3 James A. Baker III Institute for Public Policy, Rice University, Houston, Texas, United States of America

*At the end of the day, there is no handbook for the evolving US–China relationship. But the stakes are much too high for us to fail.*

—United States Secretary of State Hilary Clinton [1]

A 2012 joint survey of international relations scholars at universities in the United States and global policymakers in the US government revealed some sharp disagreements between these two groups with respect to the priority rankings of the top foreign policy problems facing the US in the next decade and beyond [2]. Whereas the academics prioritized global climate change and the collapse of the euro, the US policymakers highlighted international terrorism and the proliferation of weapons of mass destruction [2]. Both groups, however, were in agreement that the rising power of China represents the single most formidable problem facing the US [2]. Similar sentiments were echoed by US Secretary of State Hillary Clinton, who began a key November 2011 foreign policy document entitled “America's Pacific Century" with the following statement: “The future of politics will be decided in Asia, not Afghanistan or Iraq, and the United States will be right at the center of the action" [1]. Her statement also highlights China's special role in American foreign policy and the urgency for the US and China “to work together to ensure strong, sustained, and balanced future global growth" [1].

The global control and elimination of the world's neglected tropical diseases (NTDs) represent exciting and substantive opportunities to enhance and expand Sino–US relations. For the reasons highlighted below, the NTDs may also provide a useful framework for science diplomacy between the US and China in the coming decade.

***Both China and the US share a historical legacy of NTDs, and the populations of both countries suffered greatly from NTDs during the 20th century.*** Up until the time of its liberation in 1949, China was often known as the “sick man of Asia," referring to the nation's pervasive poverty and disease, especially hookworm and other NTDs [3]. Schistosomiasis had an especially important impact on China's modern history and may have been a factor in thwarting a communist assault to take back Taiwan. During the Cold War, schistosomes were known as the “blood fluke that saved Formosa"—and the widespread presence of this infection in the Yangtze River valley prompted the mobilization of more than a million peasants to bury or remove schistosome-transmitting snails during the Great Leap Forward [4], [5]. As late as the 1980s, a nationwide survey of almost 1.5 million people in all 30 provinces revealed that China had the world's largest number of cases of intestinal helminth infections, including more than 500 million cases of ascariasis and approximately 200 million cases each of trichuriasis and hookworm infection, in addition to almost 1 million cases of schistosomiasis [6]. Similarly, in the US during the first half of the 20th century, hookworm and other intestinal helminth infections, as well as typhoid fever and malaria, were highly endemic throughout the American South, where they hindered economic development and trapped people in poverty [7], [8]. Outbreaks of yellow fever were also common [9].

***Both China and the US made great strides in solving their own NTD problems.*** Over the past 50 years, China has made great strides in reducing the prevalence and intensity of some of its most important NTDs. For instance, through low-technology approaches directed at snail control and mass treatment (as well as overall improvements in sanitation and potable water), China reduced its schistosomiasis prevalence more than 90% from its initially documented level prior to the Great Leap Forward during the 1950s [10]. Similarly, through heroic national efforts at fortifying the salt with diethylcarbamazine citrate and mass drug administration in the decades following the Cultural Revolution, China became the first country to eliminate lymphatic filariasis, thereby paving the way for mass treatment efforts leading to global elimination [11]. Both China and the US made great progress in reducing the prevalence of hookworm and other intestinal helminth infections in the last half of the 20th century. While mass treatments of these infections undoubtedly had some role in these helminthic disease elimination efforts, the real contribution of large-scale mass chemotherapy relative to aggressive economic reforms remains unclear. Thus, China has achieved success in intestinal helminth control (primarily in eastern China) through programs of aggressive economic reform and urbanization during the last two decades [3], while the US reduced intestinal helminthiases and malaria through economic transformations of the American South, together with urbanization, beginning in the 1930s with the New Deal legislation [7], [8], [12].

**Figure 1:**
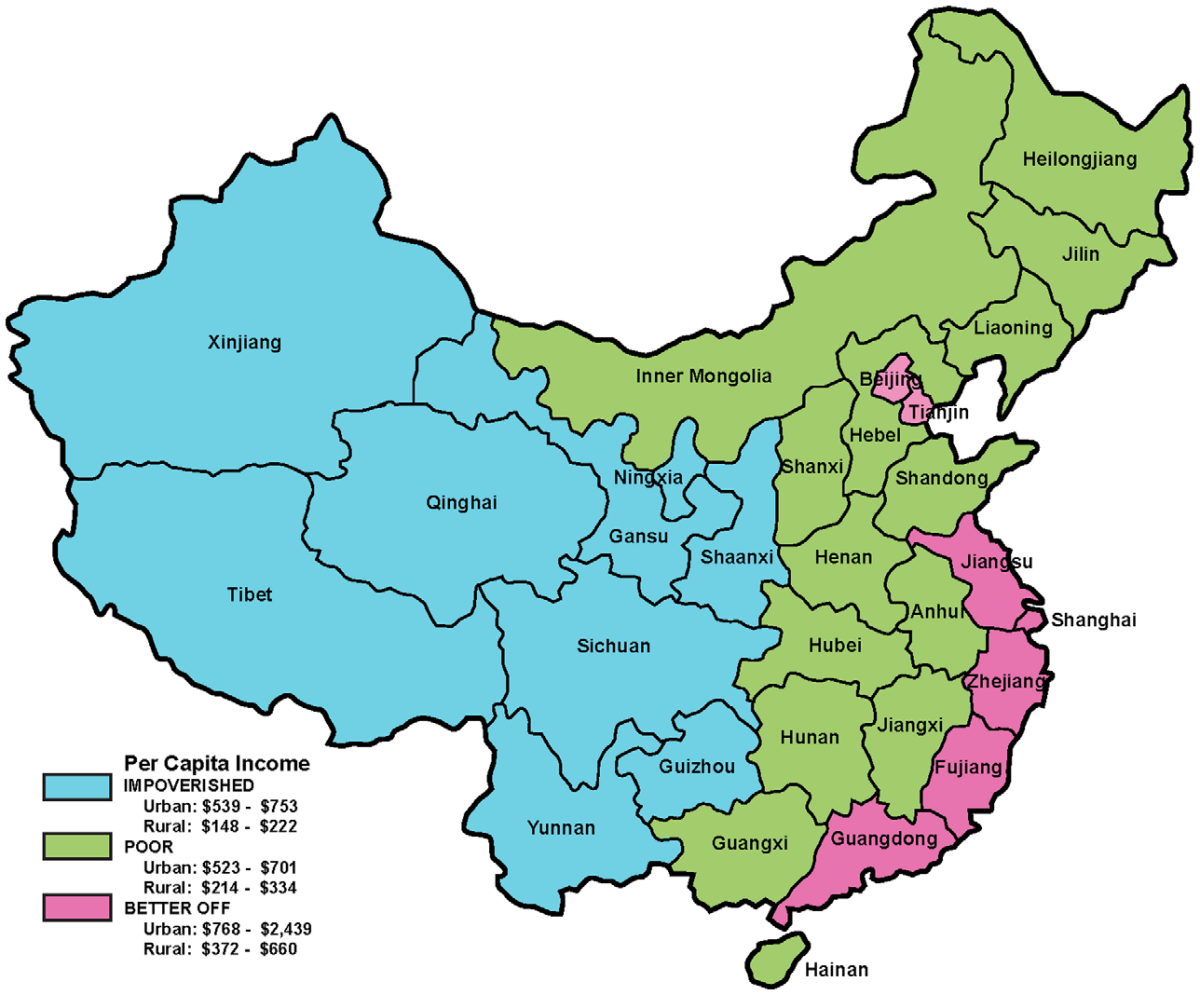
Poverty in China. Average per capita income by province. Figure modified from *Bloomberg Businessweek*, May 8, 2000. http://www.businessweek.com/2000/00_19/b3680013.htm, accessed January 19, 2012.

**Figure 2:**
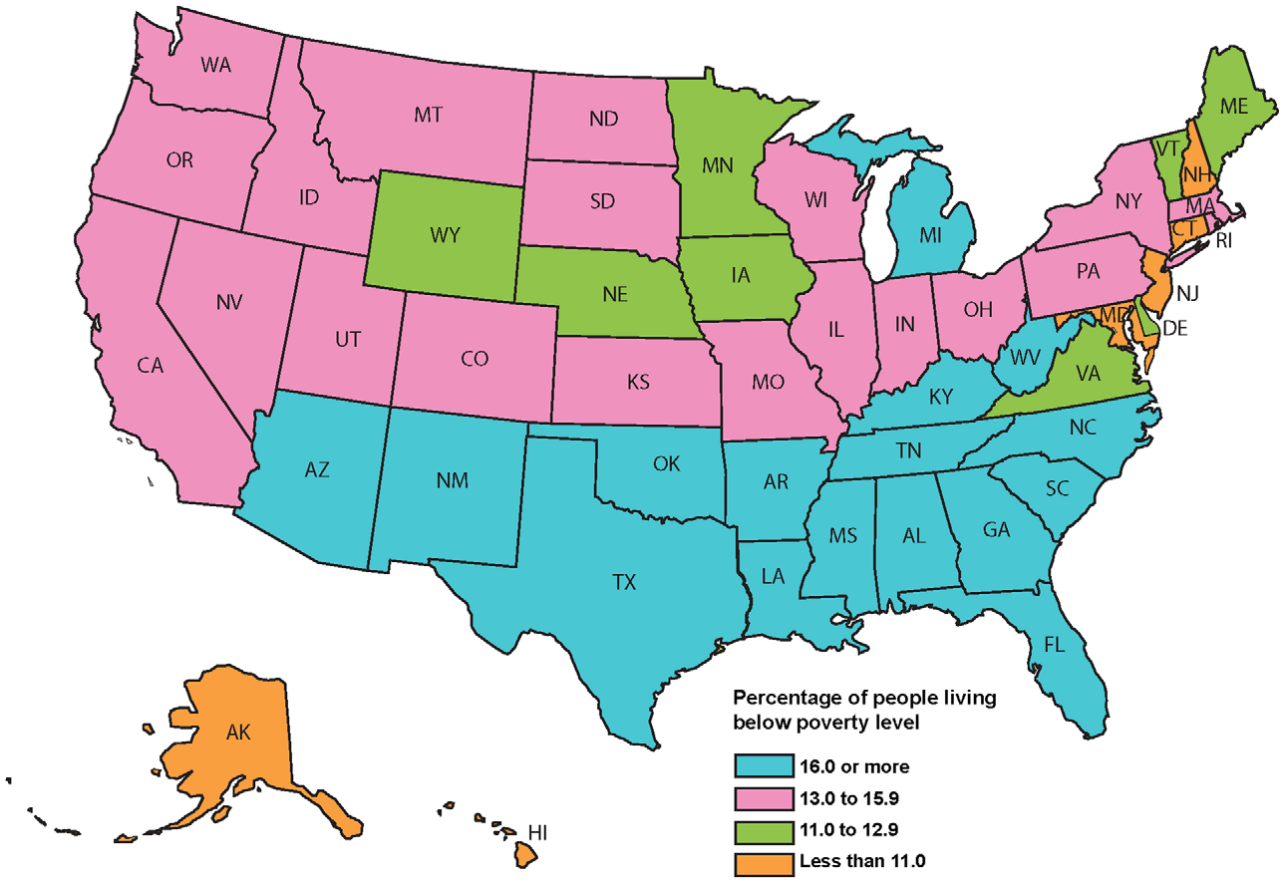
Poverty in the United States. Percentage of people in poverty in the United States in 2009 and 2010 by state. Source: US Census Bureau, 2010 American Community Survey.

***The marginalized poor living in both China and the US still suffer from surprisingly high rates of NTDs.*** Despite enormous progress in NTD control, as a nation China still has some of the largest numbers of cases of selected NTDs anywhere in the world, although in many instances overall prevalence rates are low because of the enormous population. Shown in Table 1 is a list of the major NTDs in China and the US [13]–[15]. The tens of millions of cases of intestinal helminth infections that remain are mostly concentrated in China's poorest western provinces, especially in the southwestern provinces of Guizhou, Sichuan, and Yunnan [3], [13] (Figure 1). Many of these helminth infections are hidden in remote rural and mountainous areas of these provinces [3]. Of interest is the observation that as economic development and some control measures have reduced intestinal helminth infections in eastern China, foodborne helminth infections such as clonorchiasis and echinococcosis may be emerging or on the rise [15]. Similarly, in the US there is a hidden burden of NTDs, especially in the poorest areas of Texas and the Gulf Coast [8], [16]–[18] (Figure 2). While hookworm and other intestinal helminth infections are no longer as widespread in the US as they are in China, a unique largely urban set of NTDs has arisen in their place. They include hundreds of thousands of cases of Chagas disease, cysticercosis, toxocariasis, and trichomoniasis that disproportionately affect African-Americans and Hispanics living in poverty, as well as strongyloidiasis in Appalachia [8]. Thus, in both China and the US, NTDs remain as important health disparities. In the case of China, NTDs in the southwest remain an important challenge to its health system, while in the US, NTDs in the American South and elsewhere are still largely ignored and sadly conspicuous by their absence in any meaningful debate about US health care reform.

**Table 1:** Leading parasitic and other neglected tropical diseases in China and the US.

Leading NTDs in China	Number of Cases [13]–[15]	Leading NTDs in US	Number of Cases [8]
Ascariasis	85.9 million	Toxocariasis	1.3–2.8 million
Hookworm infection	39.3 million	Giardiasis	2.0–2.5 million
Trichuriasis	29.1 million	Trichomoniasis	880,000 (African-American women)
Trachoma	27 million	Chagas disease	3,000 to >1 million
Paragonomiasis	13.8 million	Cryptosporidiosis	300,000
Clonorchiasis	12.5 million	Dengue	110,000–220,000 new infections annually
Schistosomiasis	0.7 million	Cysticercosis	41,400–169,000
Taeniasis	0.5 million	Strongyloidiasis	68,000–100,000

***Both countries have tremendous capacity for lending their expertise and financial support for global NTD control.*** An important priority for both China and the US is to aggressively pursue national efforts to eliminate their respective NTDs, which currently represent glaring health disparities. In the US, legislation was introduced in the US Congress in 2011 to begin efforts for delineating the full extent of its neglected infections of poverty [19], while the Chinese Ministry of Health last conducted a full-fledged and extensive survey of its major parasitic infections in 2005. In parallel are some important outreach efforts to control and eliminate NTDs worldwide. Through its Neglected Tropical Diseases Program, the United States Agency for International Development (USAID) of the US Department of State has provided leadership and large-scale financial support for the deployment of rapid impact packages in order to integrate the control and/or elimination of seven NTDs, including ascariasis, trichuriasis, hookworm, schistosomiasis, lymphatic filariasis, onchocerciasis, and trachoma [20], [21]. Current funding for this program may soon approach US$100 million annually in order to support more than a dozen national programs for NTD control and elimination in sub-Saharan Africa, Asia, and Latin America [20], [21]. At the same time, according to the Asian Development Bank, China's trade with Africa has increased dramatically in recent years. By 2008 it had surpassed the US$100 billion mark [22], and is expected to exceed US$110 billion in 2011 [23]. Most of Africa's exports to China are based on mineral and oil resources, especially from Angola, Democratic Republic of Congo, South Africa, and Sudan [22]. China has provided aid and invested in infrastructure in sub-Saharan Africa since the 1960s, including a railroad that links Zambia with Tanzania [24]. Despite this enormous investment in African trade and an annual economic growth of 10% or more [25], China has not yet supported NTD control and elimination in Africa. This lack of investment in disease control and elimination for Africa is especially tragic given China's extraordinary expertise and track record in NTD control and elimination at home.

***A new dialogue for NTD diplomacy.*** A joint Sino–US enterprise around NTDs and their control could be a powerful and winning combination. It could combine USAID's expertise in providing financial mechanisms and oversight in this area, as well as technical support through its public–private contractors, together with China's broad and deep expertise in parasite control linked to its history of investments in sub-Saharan Africa. Ultimately, given the current level of China's investment in Africa, it should not be onerous for China to match USAID's level of support for NTDs. Collaborating on NTD initiatives for Africa would also serve another important diplomatic purpose. It would get the two nations working together on an urgently needed peacetime project and mission, which would be both humanitarian and intellectually engaging. Cooperative efforts could include providing joint technical assistance to African health ministries in the areas of mass drug administration, integrated control and elimination through bundling of mass treatment approaches and concurrent operational research, and integrated vector management. Simultaneously, strengthening the capacity of Africa's research institutes and universities could also benefit from Sino–US scientific collaborations with African scientists. While a long-term approach to Africa's NTDs will also require economic development as it did in the US and China, a US–China NTD Initiative would nonetheless represent the very best of science diplomacy and is a project that could be initiated almost immediately.
